# TARGETING OF P21CIP1-HIGHLY-EXPRESSING CELLS ALLEVIATES NEUROPSYCHIATRIC DISORDERS WITH OBESITY

**DOI:** 10.1093/geroni/igad104.3617

**Published:** 2023-12-21

**Authors:** Lichao Wang, Binsheng Wang, Nathan Gasek, Ming Xu

**Affiliations:** UConn Health, Farmington, Connecticut, United States; UConn Health, Farmington, Connecticut, United States; University of Connecticut, Farmington, Connecticut, United States; UConn Health, Farmington, Connecticut, United States

## Abstract

Obesity remains a major factor for diabetes associated with insulin resistance and increases the risk of neuropsychiatric disorders, such as anxiety, depression, and cognitive deficits, resulting in a shortened in lifespan and healthspan. Cellular senescence, a state of permanent cell-cycle arrest, has been recognized as an important biological process underlying obesity and obesity-associated neurological disorders. My project aims to alleviate metabolic and neuropsychiatric dysfunction in obesity by targeting of p21Cip1-highly-expressing (p21high) cells, a rarely examined senescent cell population. By leveraging a novel transgene containing a p21 promoter driving Cre, we demonstrate the accumulation of p21high cells in brain with obesity, whereas, intermittently clearing p21high cells alleviates anxiety- and depression-related behavior and improve the cognitive function for obese mice. Of note, clearance of p21high cells decreased key factors of the senescence transcriptional program in hippocampus and amygdala. A survival study shows the intermittent clearance of p21high cells extends the lifespan of obese mice fed with high-fat diet. Our findings indicate p21high cells serve as a potential therapeutic target for neuropsychiatric disorders.

